# Pathophysiology of Vascular Calcification and Bone Loss: Linked Disorders of Ageing?

**DOI:** 10.3390/nu13113835

**Published:** 2021-10-27

**Authors:** Jorge B. Cannata-Andía, Natalia Carrillo-López, Osvaldo D. Messina, Neveen A. T. Hamdy, Sara Panizo, Serge L. Ferrari, on behalf of the International Osteoporosis Foundation (IOF) Working Group on Bone and Cardiovascular Diseases

**Affiliations:** 1 Bone and Mineral Research Unit, Instituto de Investigación Sanitaria del Principado de Asturias (ISPA), Retic REDinREN-ISCIII, Hospital Universitario Central de Asturias, 33011 Oviedo, Spain; ncarrillolopez.huca@gmail.com (N.C.-L.); sarapanizogarcia@gmail.com (S.P.); 2 Investigaciones Reumatológicas y Osteológicas (IRO), Buenos Aires 1114, Argentina; drosvaldodanielmessina@gmail.com; 3 Center for Bone Quality, Division Endocrinology, Department of Medicine, Leiden University Medical Center, 2333 ZA Leiden, The Netherlands; n.a.t.hamdy@lumc.nl; 4 Service and Laboratory of Bone Diseases, Department of Medicine, Faculty of Medicine, Geneva University Hospital, 1211 Geneva, Switzerland; serge.ferrari@unige.ch; 5 International Osteoporosis Foundation, 1260 Nyon, Switzerland; dominique.pierroz@osteoporosis.foundation

**Keywords:** osteoporosis, vascular calcification, mineral bone disorders, bone loss, bone fractures, fracture risk

## Abstract

Vascular Calcification (VC), low bone mass and fragility fractures are frequently observed in ageing subjects. Although this clinical observation could be the mere coincidence of frequent age-dependent disorders, clinical and experimental data suggest that VC and bone loss could share pathophysiological mechanisms. Indeed, VC is an active process of calcium and phosphate precipitation that involves the transition of the vascular smooth muscle cells (VSMCs) into osteoblast-like cells. Among the molecules involved in this process, parathyroid hormone (PTH) plays a key role acting through several mechanisms which includes the regulation of the RANK/RANKL/OPG system and the Wnt/ß-catenin pathway, the main pathways for bone resorption and bone formation, respectively. Furthermore, some microRNAs have been implicated as common regulators of bone metabolism, VC, left ventricle hypertrophy and myocardial fibrosis. Elucidating the common mechanisms between ageing; VC and bone loss could help to better understand the potential effects of osteoporosis drugs on the CV system.

## 1. Introduction

**Figure 1:**
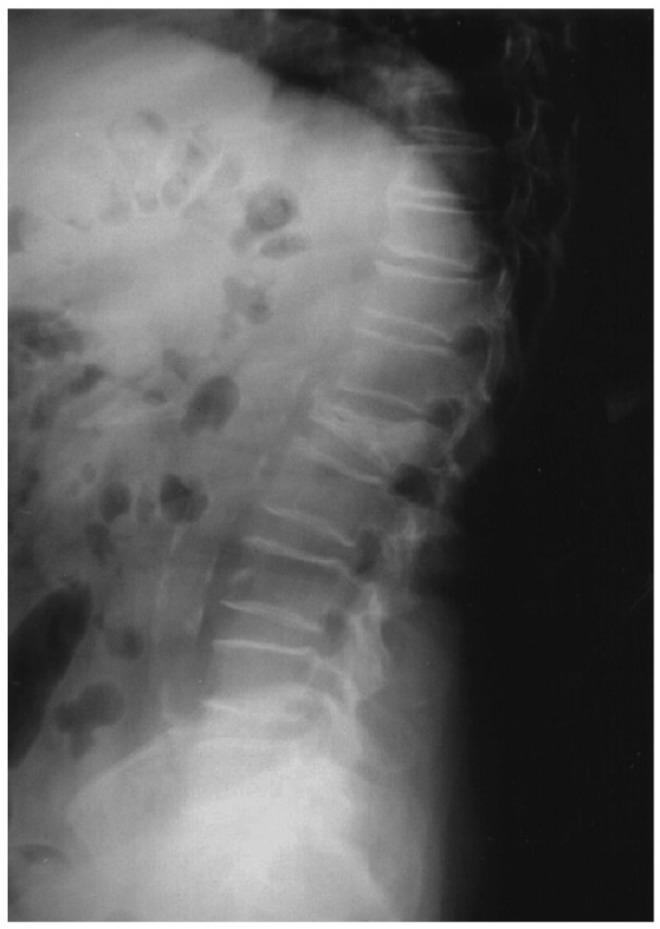
X-ray image of a 71 years old woman with aortic calcifications, low bone density and a vertebral fracture.

Vascular calcification (VC), bone loss and increased fracture risk are frequent age-associated disorders [1,2,3,4,5,6,7,8]. A good example of this association is depicted in Figure 1, which shows a 71 years old woman with aortic calcifications, low bone density and a vertebral fracture, a common observation in clinical practice. Unfortunately, such observations are too often considered as “physiological” ageing [7] rather than pathophysiological processes with potentially common mechanisms. Optimal vascular function relies on optimal maintenance of blood pressure and remodelling of the extracellular matrix of blood vessels. These functions are accomplished by vascular smooth muscle cells (VSMCs) of the physiological contractile phenotype.

VC is an active tightly regulated process driven by trans-differentiation of the physiological contractile VSMCs within the vascular wall into a “calcific” osteogenic/osteoblastic phenotype, with the phenotype-switching leading to the deposition of hydroxyapatite mineral in the intimal and medial layers of the arterial wall associated with increased risk for cardiovascular disease [9]. Although both intimal and medial calcifications of the vascular wall are primarily initiated by osteogenic/osteoblastic VSMCs, there are clear differences in their pathologic features, driving factors and differential deleterious effects on the cardiovascular system leading to arterial stiffness, ventricular dysfunction, heart failure and obstrucive disorders such as myocardial infarction and stroke [10].

Epidemiological evidence has been mounting over the past three decades for the co-existence of vascular calcification, bone loss and increased fracture risk in the ageing population, but also in chronic diseases such as diabetes and chronic kidney disease (CKD), in which the cardiovascular system and the skeleton undergo a process of accelerated ageing. Few pioneering studies described that aortic calcification, osteoporosis, bone loss and ageing were associated processes [1,2,3,4]; later on, some studies suggested that other factors, beyond ageing, may play a role in these concomitant processes [5,6,7,11,12].

One of them, based on a cohort study of postmenopausal women followed for almost 2 years, showed that the progression of VC was more pronounced in women who experienced a greater bone loss, independently of age [6]. Similar results were observed in a randomly selected sub cohort of the EVOS-EPOS study, in whom after 4 years of follow-up, women with more severe aortic calcification showed a greater decrease in bone mass and a higher incidence of new bone fragility fractures [7]. Similar results were also described in a large cohort of haemodialysis patients followed during 2 years [11], and in diabetic patients [13]. Blood supply differences between youth and senescence [14], and atherosclerotic disease could play a role [15]. In addition, coronary and aortic calcification and vascular stiffness have been found to be associated with low bone turnover and bone loss [16,17,18]. In fact, it has been suggested that the latter should be considered a risk factor for coronary arterial disease [19]. 

**Figure 2:**
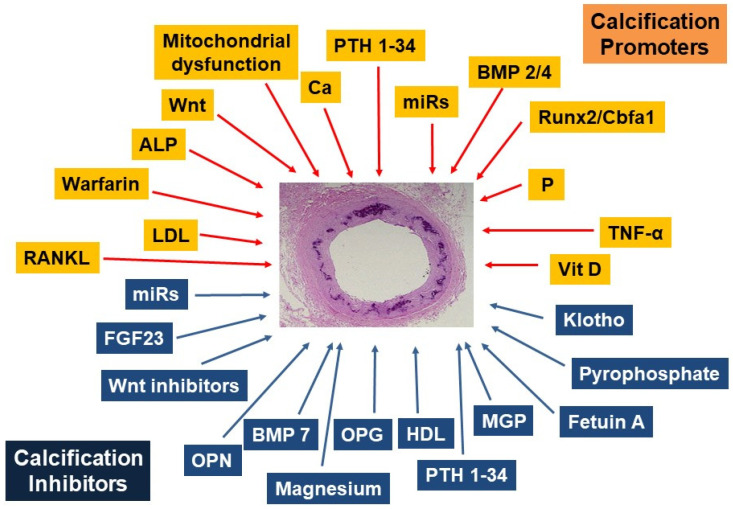
Promoters and inhibitors of vascular calcification. RANKL, receptor activator of nuclear factor-kappa B Ligand; LDL, low-density lipoprotein; ALP, alkaline phosphatase; Ca, calcium; BMP, bone morphogenetic proteins; P, phosphate; TNF-α, tumor necrosis factor-alpha; Vit D3, calcitriol; MGP, matrix GLA protein, HDL, High-density lipoprotein; OPG, osteoprotegerin; OPN, osteopontin; FGF23, fibroblast growth factor 23. (Modified with permission of Oxford University Press from [12]).

Hence, an important question arises about whether the process of ageing favours the deposition of calcium in vessels instead of bones, and/or whether reduced or accelerated bone turnover can trigger calcification of the arterial wall? Recent experimental data suggest that the association between VC and osteoporosis could result not only from ageing itself, but also from common factors that can simultaneously promote progression of VC and bone loss. In fact, the majority of factors implicated in the process of VC are molecules associated with bone metabolism [12,20,21,22] (Figure 2). The study of the signals and mechanisms involved in the crosstalk between vessels and the skeleton is a topic of intensive research due to its high clinical relevance, as discoveries in this area might improve the current therapeutic strategies used in the management of vascular and bone disorders. In this review, the first of a series of two, we will mainly concentrate on the pathogenesis of VC, discussing the most plausible links of this disorder with bone loss. In the second review of this topic, will be focused on the clinical aspects of the relationship between cardiovascular disorders and atherosclerotic diseases with bone loss and fragility fractures.

## 2. Pathophysiology of Vascular Calcification

VC is an active process of precipitation of calcium and phosphate as a consequence of the unstable super-saturation of the exchangeable calcium and phosphorus pools. The process involves a transition of the vascular smooth muscle cells (VSMCs) in the vascular walls. The VSMC cells undergo a transition away from their mesenchymal contractile functional state to a mesenchymal secretory osteoblastic phenotype [20,23]. In recent years the molecules involved in the change of the VSMC phenotype have been extensively studied, the evidence suggests that these changes are driven by factors that promote and/or inhibits VC. Several of these calcification promoters and inhibitors have been identified, some of which may act systemically and/or locally (Figure 2) [12].

The osteoblastic transition is followed by the osteoblast-like VSMC release of cell-derived matrix vesicles that contain hydroxyapatite and finally the full loss of their muscular phenotype [24]. As mineralization takes place, a macroscopic consequence in large and medium-caliber arteries is an increased stiffness, which increases the relative risk of mortality in the general population, diabetics and CKD patients [5,11,16]. These VSMC osteoblast-like cells express markers of bone formation and generate calcium-phosphorus deposits in the vasculature analogous to those mediating skeletal calcification [20,21,24,25]. The analogy to bone formation is particularly evident in the atherosclerotic calcification of the neo-intima that occurs in several inflammatory diseases, even though in the latter, medial arterial calcification is the most prevalent form of VC. The decrease of normal inhibitors of calcification, the increase of promoters and the release of exosomes plays a major role, particularly evident in CKD [25].

In fact, VC seems to be a response to ageing and other conditions, such as the uremic environment, in which there is a loss of VC inhibitors such as, fetuin A, pyrophosphate (PPi), osteopontin, matrix-Gla protein [26,27], all inhibitors of the hydroxyapatite formation. Also the mitochondrial dysfunction in calcifying VSMCs with decreases in MMP/ATP production and excessive mitochondrial fission [28] may play a role. All these factors together with the “de novo” VSMC expression of skeletal transcription factors such as CBFA-1, (known also as RUNX2), MSX2 and SOX9 [29,30], bone morphogenetic proteins (BMPs) such as BMP2 and BMP4, and bone forming proteins, such as tissue-nonspecific alkaline phosphatase (TNAP) and osteocalcin, are key for the osteoblast differentiation (Figure 2). TNAP, expressed in VSMC osteoblast like cells, hydrolyses PPi a major determinant of hydroxyapatite formation in bone and vessels [31]. Osteocalcin, currently used as a marker of bone activity is produced by osteoblasts and VSMC osteoblast-like cells and stored in the mineralized matrix [32,33,34]. When osteocalcin is overexpressed in VSMCs, it shifts cells towards enhancing the uptake of glucose and also stimulates calcification [35].

## 3. Pathophysiology of Bone Loss in Osteoporosis

Osteoporosis is a systemic skeletal disorder characterized by loss of bone mineral and microstructural alterations in the trabecular and cortical compartments, leading to decreased bone strength. The mechanisms by which bone loss occurs are well understood, including the role of pro-inflammatory cytokines such as TNF alpha, IL-1 and IL-6 on the activation of bone resorption and the inhibition of bone formation [36]. These cytokines are also involved in VC [37,38].

**Figure 3:**
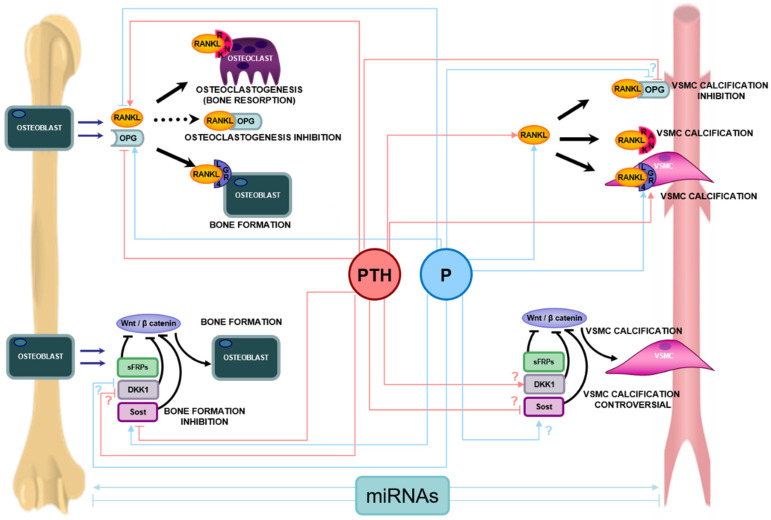
Main pathways of bone metabolism (RANK-RANKL-OPG-LGR4 System and Wnt/β-catenin) and their involvement in vascular calcification, and main effects of parathyroid hormone (PTH) and phosphorus (P) on both pathways. In bone, the osteoblast synthesizes and secretes RANKL and OPG. The binding of RANKL to RANK in osteoclast precursors induces their activation, maturation and survival, and therefore osteoclastogenesis and bone resorption. The osteoblast also synthesizes OPG that prevents the RANKL-RANK union, inhibiting osteoclastogenesis. In addition RANKL can bind LGR4 triggering bone formation signals and bone mineralization. In vascular smooth muscle cells (VSMC), the binding of RANKL to both RANK and LGR4 induces mineralization signals and vascular calcification. The union of RANKL to OPG, prevents calcification of VSMC. The Wnt/β-catenin’s pathway activation promotes the transcription of bone forming genes, regulating pre-osteoblast differentiation and osteoblast activity. The Wnt/β-catenin pathway has several inhibitors such us Dickkopf1 (Dkk1), sclerostin (Sost), and the secreted Frizzle related proteins (sFRPs), which are able to block the Wnt/β-catenin pathway, inhibiting the osteoblast differentiation and survival. The Wnt/ß-catenin pathway is also involved in the process of vascular calcification, though there is still controversy regarding the regulation of inhibitors of the Wnt/ß-catenin pathway in the process of vascular calcification. MicroRNAs (miRNAs) can regulate bone formation and/or resorption and mineralization but also can promote or inhibit VSMC calcification. Pointed arrow means activation and stop arrow means inhibition or blockage. In case of controversy in the literature, the most accepted option is included in the figure accompanied by a “?” symbol.

Among them, the binding of receptor activator of nuclear factor-kappa B (RANK) Ligand (RANKL) to its receptor RANK on osteoclasts progenitors, which triggers osteoclasts differentiation and activation, plays a prominent role in osteoporosis but also in VC [39]. Loss of estrogen during menopause leads to an increased expression of RANKL [40] and a decreased expression of osteoprotegerin (OPG), its natural antagonist, by bone cells (including osteoblasts, osteocytes and T lymphocytes), thereby increasing bone resorption in all compartments [39]. In turn, the RANKL antagonist denosumab is a potent inhibitor of bone resorption used for the treatment of both osteoporosis and bone metastases [41,42] (Figure 3).

Equally important in the process of bone fragility is the role of bone formation by osteoblasts that occurs in response to bone resorption, i.e. a remodeling process, and to mechanical forces, i.e. a modelling process. Whereas the former occurs at endosteal surfaces, the latter occurs predominantly on periosteal surfaces and is predominantly controlled by sclerostin, which is expressed by osteocytes and acts as an inhibitor of the Wnt/ß-catenin pathway that is a potent stimulus for the differentiation of bone forming cells. Again this pathway also plays a role in the pathogenesis of VC [43] (Figure 3). Although it is not yet clear what the role of sclerostin is in osteoporosis, in particular whether its levels in bone are higher and/or less responsive to mechanical forces with ageing, the inhibitors of sclerostin, particularly romosozumab, potently increase bone mass and decrease fracture risk in osteoporosis [44].

## 4. Role of Key Regulators of Bone Metabolism on VC

### 4.1. Parathyroid Hormone and FGF23

Parathyroid hormone (PTH) plays a key role not only on serum calcium homeostasis and bone turnover but also on VC through several direct and indirect actions, including the regulation of the RANK/RANKL/OPG system and of the Wnt/ß-catenin pathway [45,46] (Figure 3). The direct effects of PTH on osteoblasts and osteocytes, and indirect effects on osteoclasts, promote bone formation and bone resorption. In addition, PTH modulates the role of several VC promoters, such as calcium, phosphorus, and vitamin D [47].

High PTH induces high bone turnover and it has been frequently associated with extensive VC [18], although its role in the latter is still controversial. While some authors found that PTH 1–34 inhibited calcification [48], others found that PTH 7–84 fragments increased VC [49]. It has been shown that PTH alone is not able to induce VC, the presence of at least normal calcium and phosphorus are needed [50]. Under such conditions, the expose to different PTH 1–34 concentrations (10^−11^ M to 10^−6^ M), showed a U-shaped relationship with VC. Low PTH 1–34 concentration, in the range of 10^−11^ M to 10^−8^ M reduced, while high PTH concentrations (>10^−7^ M) increased the VSMC calcium deposition and the expression of osteogenic genes [51]. In addition, high serum phosphorus further elevated the VC of VSMCs induced by high PTH [21,51,52]. Moreover, the silencing of PTH1R, the most abundant PTH receptor in VSMC, partially abolished the pro-calcifying effect of high PTH demonstrating a PTH/PTH1R-driven induction of VSMCs calcium deposition [51,52].

The two phosphaturic hormones, PTH and FGF23, may independently contribute to the development and progression of VC [53,54], because as mentioned, VSMCs express PTHR1, so they might be susceptible to regulation by PTH. Interestingly, PTH actions on bone and the vasculature would thus be opposite to those of fibroblast growth factor 23 (FGF23). In bone, FGF23 induction of Dkk1 expression, would adversely impact the Wnt/ß-catenin pathway favoring bone loss, whereas, Dkk1 induction, if present in blood vessels, could attenuate VC [46].

### 4.2. The Role of Phosphorus

Phosphorus is an essential component of hydroxyapatite. Whereas low phosphorus levels lead to poor mineralization, the excess of phosphorus causes a large number of multifaceted adverse consequences on mineral homeostasis, negatively impacting on bone and vascular health and survival in the general population [55]. The risk of an excess in phosphorus consumption is becoming a health threat due to its silent contribution in occidental diets rich in organic phosphorus and to the generalized use of food preservatives [56]. The retention and accumulation of phosphorus exert direct pro-ageing actions accelerating renal, bone and cardiovascular damage [57]. As phosphorus is a potent stimulator of PTH secretion, it is very difficult in the presence of high serum levels of phosphorus to discriminate between the actions of high PTH and those attributable to high phosphorus [51].

The clinical impact of high phosphorus itself on bone metabolism is still controversial. Clinical and experimental studies have shown that hyperphosphataemia was associated with increased risk fracture in general population [58] and significant reduction in bone strength in normal rats [59], likely facilitated by increases in PTH. Conversely, in vitro studies have shown that high phosphorus stimulate osteoblast proliferation and differentiation, osteocyte maturation and matrix formation and reduces the expression of RANKL, inhibiting osteoclastogenesis [60,61,62,63,64] (Figure 3).

By contrast, the role of high phosphorus in VC has been more clearly established. High phosphorus is a potent systemic promoter of VC by stimulating VSMCs transition to osteoblastic phenotypes. The silencing of the putative phosphorus channel, the sodium-dependent phosphorus co-transporter, Pit-1, inhibit the phosphorus-stimulated mineralization of VSMCs [65], indicating that VC can be regulated by the cellular uptake of phosphorus in these cells. In addition, Intracellular phosphorus increases hydrogen peroxide and directly activate the AKT pathway, increasing RUNX2, the transcription factor which drives the expression of the osteoblast transcriptome and stimulates the release of matrix vesicles. High phosphorus can also influences the levels of several microRNAs (miRNAs), critical for vascular health, hence impacting on the process of VC [66].

It is traditionally accepted that VC is driven by intracellular increments in phosphorus, transported to the matrix as hydroxyapatite by calcifying VSMCs to produce mineralized areas in the vasculature. In addition, phosphorus is able to interact with calcium at physiological concentrations, forming passively calcium-phosphorus deposits. Thus, VC may also occur as a consequence of the loss of the ability of VSMCs to inhibit mineralization. Furthermore, it has been suggested that “per se”, the deposited mineral may favor the transition of VSMCs to bone-forming phenotype [25,31].

### 4.3. The RANK/RANKL/OPG System

In the mid-1990s, the RANK/RANKL/OPG pathway was discovered as a fundamental regulator of bone modeling [67]. Although its role in skeletal maintenance is well known, several studies have also shown it plays a role in the calcification of VSMCs [68,69] (Figure 3).

Although OPG is a typical bone protein, it is also expressed in the media of large arteries in VSMCs [70] and in other cells types of these vessels such as endothelial cells [71,72]. OPG acts as a soluble inhibitor that prevents RANKL binding and the subsequent stimulation of its receptor RANK [73]. The OPG knockout mouse presents osteoporosis and severe calcifications of the aorta and renal arteries suggesting that this system is involved in VC [70,74]. Moreover, RANKL and RANK have only been found in the calcified areas of arteries of transgenic mice, but not in the arteries of wild type mice [75]. RANKL induces calcification of VSMCs in vitro inducing the expression of BMP4 [68]. In vivo, RANKL transgenic mice also develop ectopic calcifications, including the heart, although it has not been looked carefully into the vascular wall [76]. OPG treatment has thus been shown to prevent the VC induced by both, vitamin D and warfarin in rats, and can also prevent VSMCs calcification in vitro [68]. All these aspects support the involvement of the RANK/RANKL/OPG axis in VC. 

The discovery in 2016 of a new receptor for RANKL, the leucine-rich repeat-containing G-protein-coupled receptor 4 (LGR4) [77], also called GPR48, which counteracts RANKL-driven osteoclastogenesis and is also an inducer of Wnt/ß-catenin pathway [78], provides a novel candidate to link bone formation with VC. LGR4 extracellular domain binds RANKL precluding RANKL-RANK binding and induces the expression of bone-related genes such as Runx2 and osteocalcin [77,79]. Recent studies in uremic rats fed a high P diet have shown that LGR4 aortic expression markedly increased in response to high PTH levels. Importantly, deletion of the LGR4 gene in VSMCs completely prevented PTH-induced VC [52]. The evidence demonstrating the positive role of LGR4 stimulating osteoblast activity and bone formation could support the possible implication of LGR4 in the process of VC, where VSMCs undergo a phenotypic transformation to osteoblast-like cells [52].

RANKL and OPG expression is also regulated in osteoblast by other factors such as vitamin D, calcium, TNF-α, glucocorticoids, prostaglandins, and several interleukins (IL) [80]. The latter, reveals the importance of immune factor’s in the regulation of mineralization signals. In fact, a term is used to describe this topic, “Immunoporosis” (immunology of osteoporosis), where T cells have special relevance [81]. Activated helper T cells, (Th) especially the Th17 subpopulation, are sources of RANKL responsible of bone resorption mainly through IL-17 [82]. Thus, Th cells are therapeutic targets for the bone destruction associated with T cell activation in inflammatory processes and it could act as a link between bone loss and VC. The bone RANKL/OPG ratio is a recognized biomarker of the degree of bone remodeling and bone mass [83]. However, there is to date no consensus on the accuracy of the RANKL/OPG ratio in estimating the risk of VC [84].

### 4.4. The Wnt/ß-Catenin Pathway

The Wnt/ß-catenin pathway play an important role not only in normal bone formation [46,85] but also in VC [21,51,86]. Preventing the inhibition of Wnt/ß-catenin pathway in bone is one of the most promising therapeutic targets to promote new bone formation [87] (Figure 3). 

In a study in diabetic rats with chronic renal failure, using neutralizing monoclonal antibodies against Dkk1, another Wnt/ß-catenin inhibitor, was sufficient to prevent bone loss without having an adverse impact on the vasculature [88]. In contrast, other study in rats with chronic renal failure and aortic calcification showed an increase in gene expression of several inhibitors of the Wnt/ß-catenin pathway, such as the secreted Frizzled related proteins (SFRPs) 1, 2 and 4 suggesting that inactivation of Wnt/ß-catenin pathway in the vessel may provide a local protective mechanism against the progression of VC [21]. Interestingly, in vitro studies have shown the decrease in Wnt/ß-catenin inhibitors, such as SFRPs, that is associated with greater calcification could be compensated by an increase in other Wnt/ß-catenin inhibitors to balance the system [89,90,91].

The inhibition of sclerostin in bone by intermittent PTH administration partly mediates PTH anabolic effects, but it will be critical to examine whether PTH-induced reduction of sclerostin in vessels favors Wnt/ß-catenin-driven VC. Indeed, recent studies in uremic rats comparing the impact of elevated and normal PTH levels (achieved through parathyroidectomy and PTH 1-34 supplementation), demonstrated for the first time an effect of high PTH on VC independent of hyperphosphatemia [51], which was corroborated in vitro. Indeed, dose response studies to PTH in VCMCs supported the direct calcifying properties of high PTH and also the protective actions of low PTH despite a similar pro-calcifying environment [51].

### 4.5. The Role of microRNAs in Bone and Vascular Metabolism

Micro RNAs (miRNAs) are small single-stranded non-coding RNAs that mediate post-transcriptional gene silencing effects are main regulators not only of skeletal related genes but also of genes involved in cardiovascular complications, as shown for VC [66,92,93] (Figure 3), left ventricle hypertrophy and myocardial fibrosis [94,95,96].

Skeletal development is a multistage process in which miRNAs can regulate the bone formation/resorption remodeling processes, bone cell growth, differentiation and function playing an important role in bone physiology and pathophysiology during early and postnatal skeletal development. Relevant in vivo and in vitro studies have revealed a significant role for miRNAs in growth plate maturation (miR-140 and let-7), in osteoblast function (miR-2861, miR-3960, miR-182, miR-199, miR-214, miR-17-92 and miR-34) and in osteoclast actions (miR-223, miR-503, miR-148a, miR-125a, miR-21, miR-31 miR-155, miR-29b) [97,98]. Over last years, different studies have been conducted to investigate the differentially expressed miRNAs between osteoporosis patients and controls, with several miRNAs being evaluated for an earlier diagnosis of osteoporosis [99,100,101]. Some miRNAs, like miR-29a protects bone tissue from osteoporosis through repressing osteoclast regulators of RANKL and CXCL12, thus reducing osteoclasts differentiation [102].

The first study analyzing miRNAs-dependent progression of VC, identified miR-125b deregulation is a main determinant of the transition of human coronary artery arterioles into osteoblast-like cells by direct targeting of osterix gene. In fact, in vitro, the inhibition of miR-125b promotes alkaline phosphatase activity and matrix mineralization [93]. Several other miRNAs modulate the calcification process. MiR-34a promotes VSMCs mineralization by inhibiting cell proliferation and inducing senescence through AXL Receptor Tyrosine Kinase and Sirtuin 1 downregulation, respectively [103]. MiR-34b regulates VSMCs calcification both in vitro and in vivo, through the targeting of Notch1 gene expression, an important regulator of Matrix Gla Protein [104]. This miRNA list is expanding with new studies, miR-145, the most abundant miRNA in VSMC, is the master regulator of VSMC phenotype, reductions in aortic miR-145 occur with exposure to high phosphorus or to calcifying conditions [105]. The maintenance of vascular miR-145 levels should help to prevent/attenuate the loss of the vascular contractile phenotype and reduce the VSMC osteogenic differentiation.

Looking at the practical use of miRNAs in future, the discovery that miRNAs are stable in plasma support their potential role as biomarkers for the early diagnosis of alterations in bone and vasculature health [106]. In addition, we could hypothesize that therapeutically, once the involvement of a miRNA in a specific alteration is identified, it would be possible to either silence or overexpress it to control or modify a vascular outcome.

### 4.6. Cellular Senescence

Cellular senescence, the irreversible growth arrest of mitotic cells, is triggered by oxidative stress, telomere shortening and/or activated oncogenes. The accumulation of senescent cells within various tissues can potentially lead to biological dysfunction and manifestation of disease associated with ageing. Senescent VSMC are also involved in the development of VC, by their increased expression of inflammatory and pro-calcifying genes (RUNX-2, ALP, type I collagen and BMP-2) [107,108].

## 5. Summary and Conclusions

This review shows that VC and bone loss that often coexist in ageing individuals, share numerous pathophysiological mechanisms. In this context, PTH, the RANK/RANKL/OPG system and the Wnt/ß-catenin pathway are the most studied factors. High PTH thus increases bone resorption and bone loss, but also triggers mechanisms that favour VC involving the RANK/RANKL/OPG and Wnt/ß-catenin pathways. Furthermore, other closely related factors such as calcium, phosphate, FGF23, Klotho, vitamin D and other regulatory factors that regulate PTH render these interactions extremely complex. The presence of low or high PTH levels, and consequently of low or high bone turnover, facilitate the process of deposition hydroxyapatite in the wall of the vessels, leading to progression of VC when present for prolonged periods. The process eventually becomes severe, potentially increasing vascular molecular signals in order to reduce “bone deposition in the vessels”, which in turn could favour the reduction of normal bone formation [21,46]. Thus, in the presence of severe VC, a vicious circle may be established, further reducing bone mass.

The increase or decrease in tissue and/or serum levels of any these factors may play a pathogenic role in both bone loss and VC, and may be potentially promisingly used as a marker of bone and cardiovascular disease. However, caution should be exerted in the interpretation of these markers. For instance, whereas higher serum levels of sclerostin have been associated with VC and poor outcomes, this may not necessarily be due to a cause and effect relationship, but to a potential overproduction of sclerostin as a protective factor against VC. Similarly, serum sclerostin levels have been positively, and not negatively, associated with higher bone mass [109].

Although the pathogenesis and progression of VC and bone loss shares several common factors and pathways, it remains a “chicken-and-egg” situation, where it difficult to stablish cause and effect as to whether bone loss is driving VC or vice-versa, or whether there is a higher level of dysregulation generated by the ageing process that impacts on both tissues simultaneously, using common mechanisms.
